# Cardiovascular and renal protective role of angiotensin blockade in hypertension with advanced CKD: a subgroup analysis of ATTEMPT-CVD randomized trial

**DOI:** 10.1038/s41598-018-20874-4

**Published:** 2018-02-16

**Authors:** Shokei Kim-Mitsuyama, Hirofumi Soejima, Osamu Yasuda, Koichi Node, Hideaki Jinnouchi, Eiichiro Yamamoto, Taiji Sekigami, Hisao Ogawa, Kunihiko Matsui

**Affiliations:** 1 Department of Pharmacology and Molecular Therapeutics, Graduate School of Medical Sciences, Kumamoto University, Kumamoto, Japan; 2 Department of Cardiovascular Medicine, Graduate School of Medical Sciences, Kumamoto University, Kumamoto, Japan; 3 Health Care Center, Kumamoto University, Kumamoto, Japan; 4 Department of Sports and Life Sciences, National Institute of Fitness and Sports in Kanoya, Kanoya, Japan; 5 Department of Cardiovascular Medicine, Saga University, Saga, Japan; 6 Diabetes Care Center, Jinnouchi Clinic, Kumamoto, Japan; 7 Division of Internal Medicine & Diabetes and Endocrine, Sekigami Clinic, Yatsushiro, Japan; 8 National Cerebral and Cardiovascular Center, Suita, Japan; 9 Department of General and Community Medicine, Kumamoto University Hospital, Kumamoto, Japan

## Abstract

The ATTEMPT-CVD study was prospective randomized active-controlled trial and the main findings had been reported. According to baseline GFR and albuminuria categories, we divided the patients of the ATTEMPT-CVD study into 2 subgroups: (Group 1) the patients with at least one of eGFR of <45 ml/min per 1.73 m^2^ and UACR of ≥300 mg/g creatinine, defined as G3b and/or A3; (Group 2) the patients except for Group 1, defined as the other patients. In patients with G3b and/or A3, the incidence of cardiovascular events was significantly less in ARB group than in non-ARB group (11 vs 22, respectively) (HR = 0.465: 95%CI = 0.224–0.965; P = 0.040). UACR was significantly less in ARB group than in non-ARB group during follow-up period in patients with G3b and/or A3 (P = 0.0003), while eGFR, plasma BNP levels, and blood pressure were comparable between ARB and non-ARB groups. Allocation to ARB therapy was a significant independent prognostic factor for cardiovascular events in patients with G3b and/or A3 (P = 0.0268). On the other hand, in the other patients, the occurrence of cardiovascular events was comparable between ARB and non-ARB groups. In patients with advanced CKD, ARB-based therapy may confer greater benefit in prevention of cardiovascular events than non-ARB therapy.

## Introduction

We performed a trial of telmisartan prevention of cardiovascular disease (ATEMPT-CVD)^1,2^ to compare the effects of angiotensin II receptor blocker (ARB)-based antihypertensive therapy and those of non-ARB antihypertensive therapy on biomarker level changes and the incidence of cardiovascular and renal events in Japanese hypertensive patients who had at least one of cardiovascular risk factors (type 2 diabetes, cerebrovascular, cardiac or peripheral arterial disease, or renal impairment). ATTEMPT-CVD study provided the evidence that ARB (telmisartan)-based antihypertensive therapy caused a greater decrease in urinary albumin/creatinine ratio (UACR) and a smaller increase in plasma BNP than non-ARB antihypertensive therapy, under similar blood pressure control^1^. However, in spite of more benefit in UACR and plasma BNP changes by ARB therapy than by non-ARB therapy, the incidence of cardiovascular and renal events did not differ between ARB group and non-ARB group^1^. Thus, the positive effects of ARB therapy on UACR and plasma BNP were not associated with cardiovascular outcome in overall hypertensive patients enrolled in the ATTEMPT-CVD study.

Patients with chronic kidney disease (CKD) are at risk of not only end-stage kidney disease (ESKD) but also cardiovascular disease and death^3^. Glomerular filtration rate (GFR) level is most often used for definition and classification of CKD^3–5^, and most of previous clinical trials on CKD patients have defined CKD based on GFR category. GFR category is divided into 5 stages (≥90 [G1], 60–89 [G2], 30–59 [G3], 15–29 [G4], and <15 [G5] ml/min per 1.73 m^2^)^3–5^. Very importantly, stage G3 is subdivided into G3a (eGFR of 45–59 ml/min per 1.73 m^2^) and G3b (GFR of 30–44 ml/min per 1.73 m^2^)^4,5^. This subdivision of G3 stage by applying a cut-off value of 45 ml/min per 1.73 m^2^ is very critical for appropriate risk assessment of ESKD and cardiovascular disease, because the patients with GFR of <45 ml/min per 1.73 m^2^ have much higher risk for cardiovascular death, ESKD and all-cause death than those with GFR of ≥45 ml/min per 1.73 m^2^ ^3–5^. However, there is no sufficient evidence from large-scale randomized trial addressing appropriate antihypertensive strategy in patients with GFR category of <45 ml/min per 1.73 m^2^ or worse stage. Besides GFR, the level of albuminuria is established to predict the prognosis of CKD progression and cardiovascular disease and death, independently of GFR^6–11^. KDIGO guideline highly recommend the use of both GFR and albuminuria categories for clinical decision making in CKD patients^4,5,12^. However, many previous clinical studies on CKD patients have paid attention to only GFR category but not to albuminuria category. Importantly, of albuminuria categories, UACR of ≥300 mg/g creatinine (macroalbuminuria), namely category A3, is established to be at much higher risk for ESKD and cardiovascular disease and death, regardless of GFR stage (even in the case of GFR stage G1 or G2)^3,12,13^. Based on these evidences, patients with at least one of eGFR of <45 ml/min per1.73 m^2^ (G3b cut-off value) and UACR of ≥300 mg/g creatinine (macroalbuminuria) have much higher risk for ESKD and cardiovascular morbidity and mortality than the patients with neither eGFR of <45 ml/min per 1.73 m^2^ nor macroalbuminuria^3–5^. However, it remains to be elucidated whether renin-angiotensin system blockers exert more benefit in prevention of cardiovascular and renal events than other antihypertensive drugs in hypertensive patients with at least one of eGFR of <45 ml/min per 1.73 m^2^ and UACR of ≥300 mg/g creatinine. Therefore, in the present subanalysis, we subdivided the hypertensive patients enrolled in the ATTMPT-CVD study into two groups; (1) the patients with at least one of eGFR of <45 ml/min per 1.73 m^2^ and UACR of ≥300 mg/g creatinine, defined as the patients with G3b and/or A3 and (2) the patients with neither eGFR of <45 ml/min per 1.73 m^2^ nor UACR of ≥300 mg/g creatinine, defined as the other patients. We examined the comparative effects of ARB therapy and non-ARB therapy on the incidence of cardiovascular and renal events and biomarker changes in the subgroup patients.

## Results

### Categorization of patients according to UACR and eGFR categories at baseline

As 6 patients of 1,228 patients enrolled in the ATTEMPT-CVD study were excluded from the present subanalysis because of no availability of baseline eGFR data, 1,222 patients were included in the present subanalysis. Table 1 shows distribution of patients according to baseline eGFR and UACR categories. In patients enrolled in the ATTEMPT-CVD study, there was no patient with eGFR stage G5 and the proportion of patients with eGFR G4 stage was very small. The number of patients with at least one of eGFR of <45 ml/min per 1.73 m^2^ (G3b, G4 or G5) and UACR of ≥300 mg/g creatinine (A3 category) was 187, which were defined as patients with G3b and/or A3. The number of patients with both eGFR of ≥45 ml/min per 1.73 m^2^ (G1-G3a) and UACR of <300 mg/g creatinine (A1 or A2) was 1,035, which were defined as the other patients.

**Table 1:** Distribution of patients according to UACR and eGFR categories at baseline.

GFR categories	eGFR (ml/min/1.73 m^2^)	Albuminuria categories		
		A1	A2	A3
		UACR of <30 mg/g creatinine	UACR of 30–300 mg/g creatinine	UACR of ≥300 mg/g creatinine
G1 and G2	≥60	510 (41.7%)	327 (26.8%)	**81 (6.6%)**
G3a	45–59	109 (8.9%)	89 (7.3%)	**29 (2.4%)**
G3b	30–44	**25 (2.0%)**	**21 (1.7%)**	**20 (1.6%)**
G4	15–29	**1 (0.1%)**	**3 (0.2%)**	**7 (0.6%)**
G5	<15	**0 (0%)**	**0 (0%)**	**0 (0%)**

Categories in bold indicate patients with at least one of eGFR of <45 ml/min/1.73 m^2^ and UACR of ≥300 mg/g creatinine. Abbreviations: UACR, urinary albumin/creatinine ratio; eGFR, estimated glomerular filtration rate.

Values are the number of patients belonging to each category. Number in parenthesis indicates percentage of patients to overall patients.

### Demographic and baseline characteristics of patients with G3b and/or A3 and the other patients

As shown in Table 2, median of UACR in patients with G3b and/or A3 was 490 mg/g creatinine and that in the other patients was 20.7 mg/g creatinine (P < 0.0001 between the groups). Median of eGFR in patients with G3b and/or A3 was 52.6 ml/min per 1.73 m^2^ and that in the other patients was 72.7 ml/min per 1.73 m^2^ (P < 0.0001 between the groups). Compared with the other patients, patients with G3b and/or A3 had higher plasma BNP (P < 0.0001), were older (P = 0.0063), and had higher serum creatinine (P < 0.0001), slightly higher serum potassium (P = 0.0329), higher blood sugar (P = 0.0002), higher hemoglobin A1c (P = 0.0007), lower hemoglobin (P = 0.0004) and higher uric acid (P < 0.0001). In patients with G3b and/or A3, baseline characteristics were well balanced between ARB group and non-ARB group, and there was no statistically significant difference regarding demographic and baseline characteristics listed in Table 2. In the other patients, baseline characteristics were also well balanced between the two treatment groups and there was no significant difference regarding baseline characteristics between ARB and non-ARB groups.

**Table 2:** Baseline characteristics of patients with eGFR of <45 ml/min per 1.73 m^2^ and/or UACR of ≥300 mg/g creatinine and the other patients.

	Total patients	Overall			G3b and/or A3		The other	
		G3b and/or A3	The other	P-value	ARB	Non-ARB	ARB	Non-ARB
	(n = 1222)	(n = 187)	(n = 1035)		(n = 96)	(n = 91)	(n = 516)	(n = 519)
UACR (mg/g creatinine)	26.1 (11.1–89.1)	490 (119–957)	20.7 (10.3–54.4)	<0.0001	409 (102–914)	529 (300–974)	20.4 (10.5–50.6)	21.1 (10.1–61.6)
eGFR (ml/min per 1.73 m^2^)	71.2 (60.0–84.6)	52.6 (40.2–74.0)	72.7 (62.4–85.3)	<0.0001	51.0 (40.0–72.3)	55.3 (40.9–74.3)	72.9 (62.1–84.9)	72.6 (62.8–86.7)
BNP (pg/mL)	18.0 (9.0–36.5)	23.3 (12.0–58.4)	17.4 (8.7–34.3)	<0.0001	27.5 (13.8–70.1)	19.2 (9.6–46.6)	17.3 (8.1–34.1)	17.5 (9.1–34.8)
Age (years)	66 ± 9	68 ± 9	66 ± 9	0.0063	68 ± 9	67 ± 9	66 ± 9	66 ± 10
Male, n (%)	712 (58.3)	113 (60.4)	599 (57.9)	0.5146	60 (62.5%)	53 (58.2%)	298 (57.8)	301 (58.0)
BMI (kg/m^2^)	25.2 ± 3.8	25.4 ± 4.0	25.2 ± 3.8	0.5198	25.3 ± 3.9	25.6 ± 4.1	25.1 ± 3.8	25.3 ± 3.8
Systolic BP (mmHg)	150 ± 16	152 ± 16	150 ± 16	0.1393	151 ± 16	153 ± 15	151 ± 16	149 ± 15
Diastolic BP (mmHg)	84 ± 12	82 ± 12	84 ± 12	0.0201	81 ± 12	83 ± 13	85 ± 12	84 ± 12
Heart rate (b.p.m)	72 ± 11	74 ± 12	72 ± 11	0.0292	72 ± 11	75 ± 13	72 ± 11	72 ± 11
Diabetes mellitus, n (%)	817 (66.9)	131 (70.1)	686 (66.3)	0.3131	69 (71.9)	62 (68.1%)	340 (65.9)	346 (66.7)
Hyperlipidemia, n (%)	704 (57.6)	106 (56.7)	598 (57.8)	0.7807	54 (56.3)	52 (57.1)	298 (57.8)	300 (57.8)
Current smoker, n (%)	216 (17.7)	36 (19.3)	180 (17.4)	0.5394	17 (17.7)	19 (20.9)	90 (17.4)	90 (17.3)
Serum or plasma values								
Creatinine (mg/dL)	0.8 ± 0.2	1.0 ± 0.4	0.7 ± 0.2	<0.0001	1.0 ± 0.4	1.0 ± 0.4	0.7 ± 0.2	0.7 ± 0.2
Potassium (mEq/L)	4.3 ± 0.5	4.4 ± 0.6	4.3 ± 0.5	0.0329	4.4 ± 0.7	4.3 ± 0.6	4.3 ± 0.5	4.3 ± 0.5
Total cholesterol (mg/dL)	196 ± 36	202 ± 48	195 ± 33	0.1420	199 ± 41	204 ± 54	195 ± 34	195 ± 32
LDL cholesterol (mg/dL)	112 ± 30	114 ± 33	112 ± 29	0.5415	110 ± 31	119 ± 35	112 ± 30	112 ± 28
HDL cholesterol (mg/dL)	56 ± 14	55 ± 14	56 ± 14	0.0736	56 ± 15	53 ± 13	57 ± 14	56 ± 14
Blood sugar (mg/dL)	135 ± 56	150 ± 63	132 ± 54	0.0002	148 ± 61	151 ± 66	130 ± 51	135 ± 57
Hemoglobin A1c (%)	6.4 ± 1.2	6.7 ± 1.4	6.3 ± 1.1	0.0007	6.5 ± 1.2	6.9 ± 1.6	6.3 ± 1.1	6.3 ± 1.2
Hemoglobin (g/dL)	13.9 ± 1.6	13.5 ± 1.8	14.0 ± 1.6	0.0004	13.4 ± 1.9	13.7 ± 1.7	14.0 ± 1.6	14.0 ± 1.5
Uric acid (mg/dL)	5.3 ± 1.3	5.7 ± 1.5	5.3 ± 1.3	<0.0001	5.8 ± 1.4	5.6 ± 1.5	5.2 ± 1.3	5.3 ± 1.3

Abbreviations: G3b and/or A3, patients with estimated glomerular filtration rate of <45 ml/min per 1.73 m^2^ and/or urinary albumin/creatinine ratio of ≥300 mg/g creatinine; The other, patients with both estimated glomerular filtration rate of ≥45 ml/min per 1.73 m^2^ and urinary albumin/creatinine ratio of <300 mg/g creatinine; UACR, urinary albumin/creatinine ratio; eGFR, estimated glomerular filtration rate; BNP, brain natriuretic peptide; BMI, body mass index; BP, blood pressure; LDL, low-density lipoprotein; HDL, high-density lipoprotein; ARB, antihypertensive treatment with angiotensin II receptor blocker; Non-ARB, treatment with antihypertensive drugs except for angiotensin II receptor blocker. UACR, eGFR, and BNP are expressed as median with interquartile range. Other data are mean ± s.d. for continuous values and number (%) for categorical variables. P-value was calculated using t-test or ManWhitney test for continuous variables and χ^2^ tests for categorical variables.

Table 3 shows the proportion of baseline cardiovascular diseases of overall patients and patients with G3b and/or A3 or the other patients. Proportion of previous cardiovascular diseases was slightly less in patients with G3b and/or A3 than in the other patients (25.7% vs 33.7%; P = 0.0305), and this difference was mainly accounted for by less percentage of previous left ventricular hypertrophy in patients with G3b and/or A3 than in the other patients (8.6% vs 14.8%; P = 0.0232). The proportion of previous cerebrovascular disease and the proportion of previous peripheral artery disease were not different between patients with G3b and/or A3 and the other patients. In patients with G3b and/or A3, proportion of previous cardiovascular disease, previous cerebrovascular disease, and previous peripheral artery disease were similar between ARB and non-ARB groups. Also in the other patients, the proportion of previous cardiovascular diseases was comparable between the two treatments.

**Table 3:** Proportion of baseline cardiovascular disease of patients with eGFR of <45 ml/min per 1.73 m^2^ and/or UACR of ≥300 mg/g creatinine and the other patients.

	Total patients	Overall			G3b and/or A3		The other	
		G3b and/or A3	The other	P-value	ARB	Non-ARB	ARB	Non-ARB
	(n = 1222)	(n = 187)	(n = 1035)		(n = 96)	(n = 91)	(n = 516)	(n = 519)
Previous cardiac disease, n (%)	397 (32.5)	48 (25.7)	349 (33.7)	0.0305	27 (28.1)	21 (23.1)	173 (33.5)	176 (33.9)
Myocardial infarction, n (%)	54 (4.4)	6 (3.2)	48 (4.6)	0.3815	5 (5.2)	1 (1.1)	24 (4.7)	24 (4.6)
Angina pectoris, n (%)	114 (9.3)	18 (9.6)	96 (9.3)	0.8795	9 (9.4)	9 (9.9)	46 (8.9)	50 (9.6)
Heart failure (NYHA I/II), n (%)	43 (3.5)	7 (3.7)	36 (3.5)	0.8563	4 (4.2)	3 (3.3)	19 (3.7)	17 (3.3)
Left ventricular hypertrophy, n (%)	169 (13.8)	16 (8.6)	153 (14.8)	0.0232	9 (9.4)	7 (7.7)	81 (15.7)	72 (13.9)
Atrial fibrillation, n (%)	87 (7.1)	13 (7.0)	74 (7.1)	0.9228	7 (7.3)	6 (6.6)	35 (6.8)	39 (7.5)
Previous cerebrovascular disease, n (%)	165 (13.5)	32 (17.1)	133 (12.9)	0.1165	18 (18.8)	14 (15.4)	64 (12.4)	69 (13.3)
Cerebral infarction, n (%)	97 (7.9)	19 (10.2)	78 (7.5)	0.2218	10 (10.4)	9 (9.9)	40 (7.8)	38 (7.3)
Cerebral hemorrhage, n (%)	13 (1.1)	2 (1.1)	11 (1.1)	0.9934	2 (2.1)	0 (0)	4 (0.8)	7 (1.3)
Subarachnoid hemorrhage, n (%)	8 (0.7)	0 (0)	8 (0.8)	0.2277	0 (0)	0 (0)	4 (0.8)	4 (0.8)
Transient ischemic attack, n (%)	48 (3.9)	12 (6.4)	36 (3.5)	0.0569	7 (7.3)	5 (5.5)	16 (3.1)	20 (3.9)
Previous peripheral artery disease, n (%)	6 (0.5)	2 (1.1)	4 (0.4)	0.2188	2 (2.1)	0 (0)	2 (0.4)	2 (0.4)
Lower extremities bypass surgery or angioplasty, n (%)	3 (0.2)	1 (0.5)	2 (0.2)	0.3851	1 (1.0)	0 (0)	1 (0.2)	1 (0.2)
Ankle-brachial index of <0.9 or with intermittent claudication, n(%)	4 (0.3)	2 (1.1)	2 (0.2)	0.0535	2 (2.1)	0 (0)	1 (0.2)	1 (0.2)

Abbreviations used are the same as in Table 2. Data are number (%). P-value was calculated using χ^2^ tests.

### Incidence of cardiovascular and renal events in patients with G3b and/or A3 and the other patients

**Figure 1:**
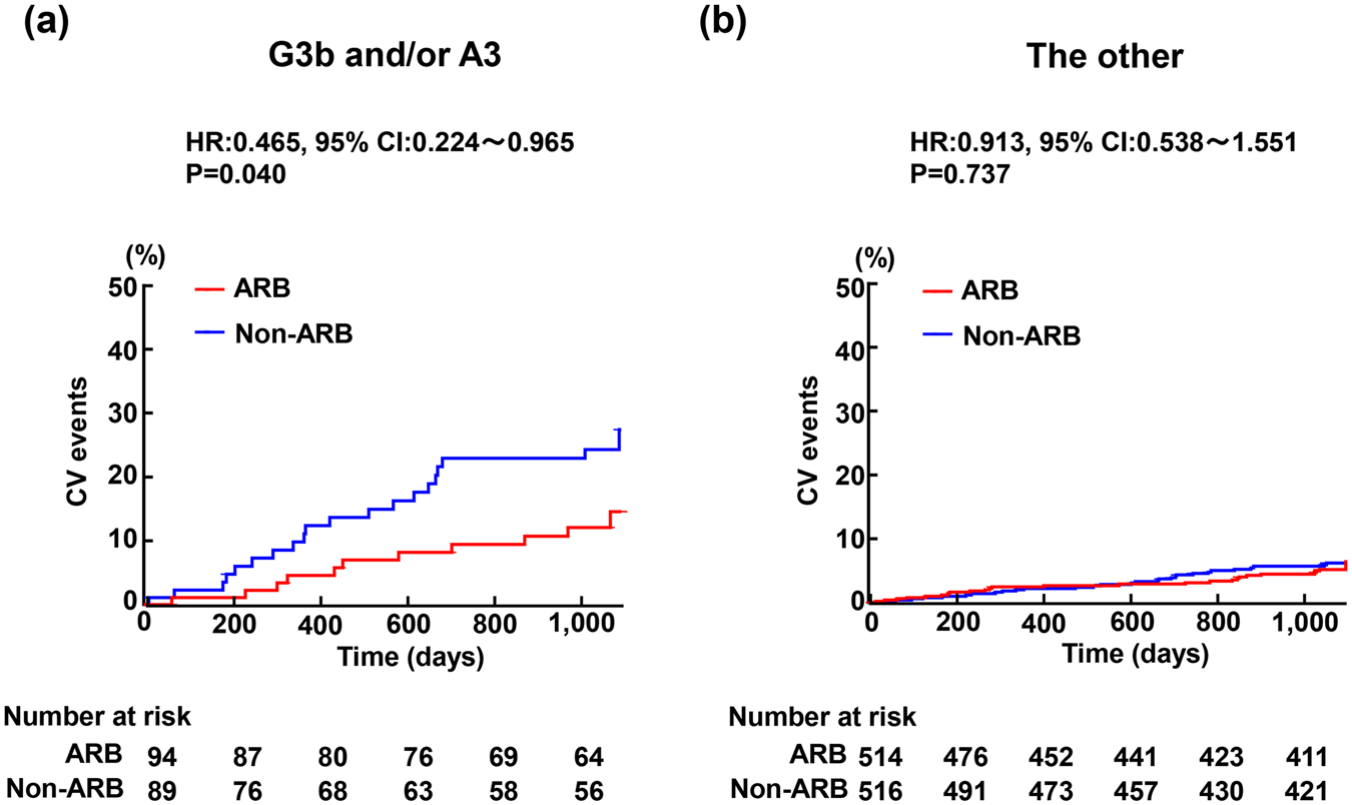
Kaplan-Meier curves for composite cardiovascular and renal events during the follow-up period in patients with eGFR of <45 ml/min per 1.73 m^2^ and/or UACR of ≥300 mg/g creatinine (**a**) and the other patients (**b**). In (**a**), the numbers of patients in ARB and non-ARB groups were 96 and 91, respectively, and the number of occurrence of endpoints was 11 and 22 in ARB group and non-ARB group, respectively. In (**b**), the number of endpoints was 27 in 516 patients assigned ARB group and 29 in 518 patients assigned non-ARB group. Abbreviations: G3b and/or A3, patients with estimated glomerular filtration rate of <45 ml/min per 1.73 m^2^ and/or urinary albumin/creatinine ratio of ≥300 mg/g creatinine; The other, patients with both estimated glomerular filtration rate of ≥45 ml/min per 1.73 m^2^ and urinary albumin/creatinine ratio of <300 mg/g creatinine; ARB, antihypertensive treatment with angiotensin II receptor blocker; Non-ARB, treatment with antihypertensive drugs except for angiotensin II receptor blocker; HR, hazard ratio; 95%CI, 95% confidence interval.

Figure 1 shows the incidence of cardiovascular and renal events in ARB group and in non-ARB group of patients with G3b and/or A3 (Fig. 1(a)) and of the other patients (Fig. 1(b)). The incidence of cardiovascular and renal events was much greater in patients with G3b and/or A3 than in the other patients. In patients with G3b and/or A3, the incidence of cardiovascular and renal events was significantly less in ARB group than in non-ARB group (HR = 0.465: 95%CI = 0.224–0.965; P = 0.040) (Fig. 1(a)). On the other hand, in the other patients, there was no significant difference regarding incidence of cardiovascular and renal events between ARB and non-ARB groups (HR = 0.913: 95%CI = 0.538–1.551; P = 0.737) (Fig. 1(b)). The P value for the treatment-subgroup interaction was 0.1397. Table 4 indicates the detail of cardiovascular and renal events occurred in ARB and non-ARB groups in patients with G3b and/or A3 or the other patients.

**Table 4:** Comparison of composite cardiovascular events between ARB and non-ARB groups in patients with eGFR of <45 ml/min per 1.73 m^2^ and/or UACR of ≥300 mg/g creatinine and the other patients.

Event	G3b and/or A3		The other	
	ARB (n = 96)	Non-ARB (n = 91)	ARB (n = 516)	Non-ARB (n = 519)
Total cardiovascular and renal events, n	11	22	27	29
Stroke, n	1	3	7	8
Transient ischemic attack, n	0	1	1	1
Sudden death, n	1	0	1	2
Acute myocardial infarction, n	2	0	3	1
Angina pectoris, n	0	1	1	4
Heart failure, n	2	4	1	2
Aortic aneurysm	0	0	1	2
Aortic dissection, n	0	1	0	0
Peripheral artery disease, n	1	2	4	3
Diabetic nephropathy, n	0	0	1	1
Diabetic retinopathy, n	1	2	5	5
Doubling of serum creatinine, n	2	8	2	0
End stage renal disease, n	1	0	0	0

Abbreviations used are the same as in Table 2.

### Time course of changes in UACR during the follow-up period

**Figure 2:**
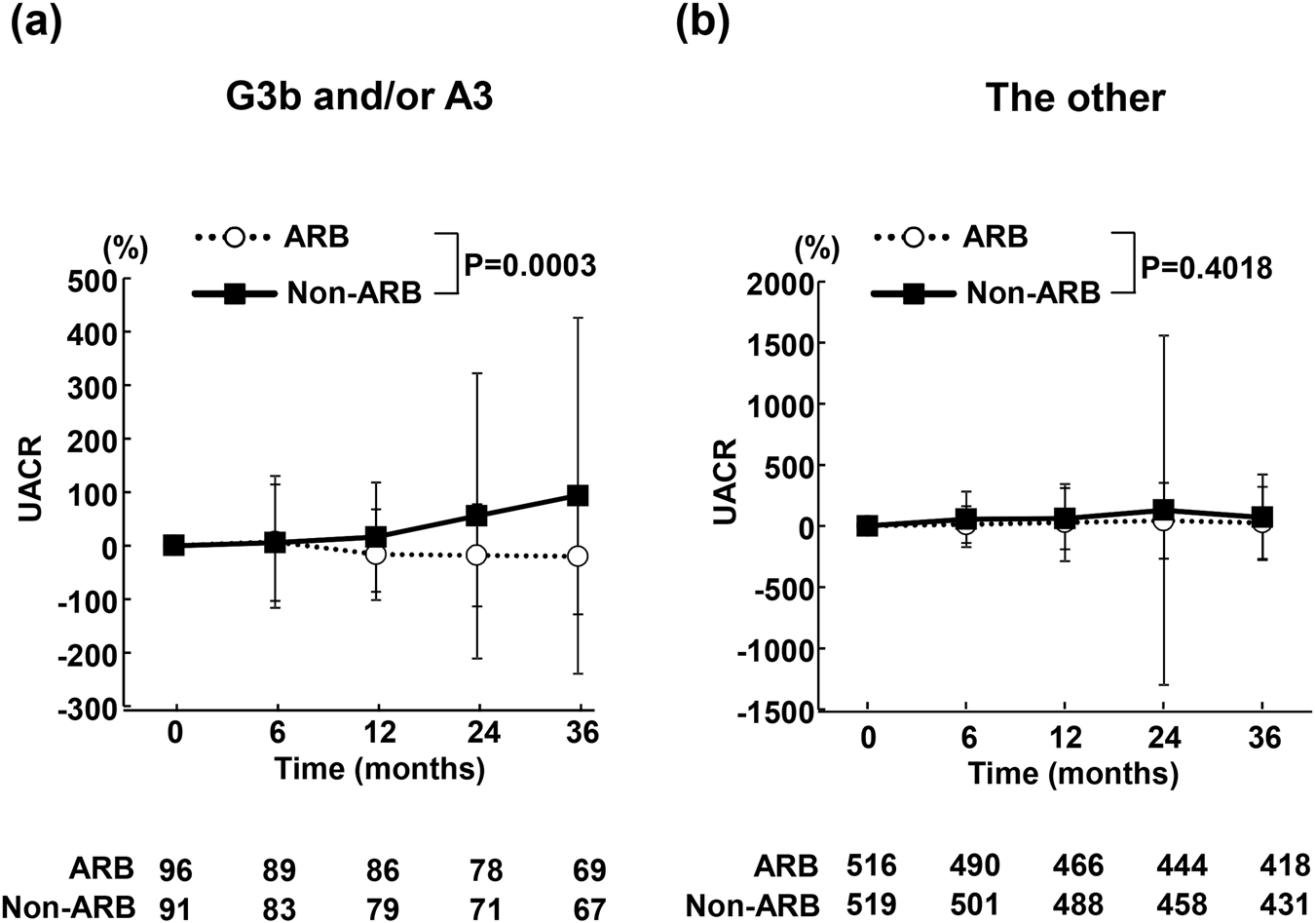
Time course of % changes in urinary albumin/creatinine ratio (UACR) in ARB and non-ARB groups in patients with eGFR of <45 ml/min per 1.73 m^2^ and/or UACR of ≥300 mg/g creatinine (**a**) and the other patients (**b**). Abbreviations used are the same as in Fig. 1. Values are mean ± SD.

Figure 2 shows the time course of changes in UACR in the patients with G3b and/or A3 and the other patients. As shown in Fig. 2(a), UACR in ARB group was significantly less than in non-ARB group during follow-up period (P = 0.0003). On the other hand, there was no significant difference in time course of UACR changes between the ARB and non-ARB groups in the other patients (P = 0.4018) (Fig. 2(b)).

### Time course of eGFR during the follow-up period

**Figure 3:**
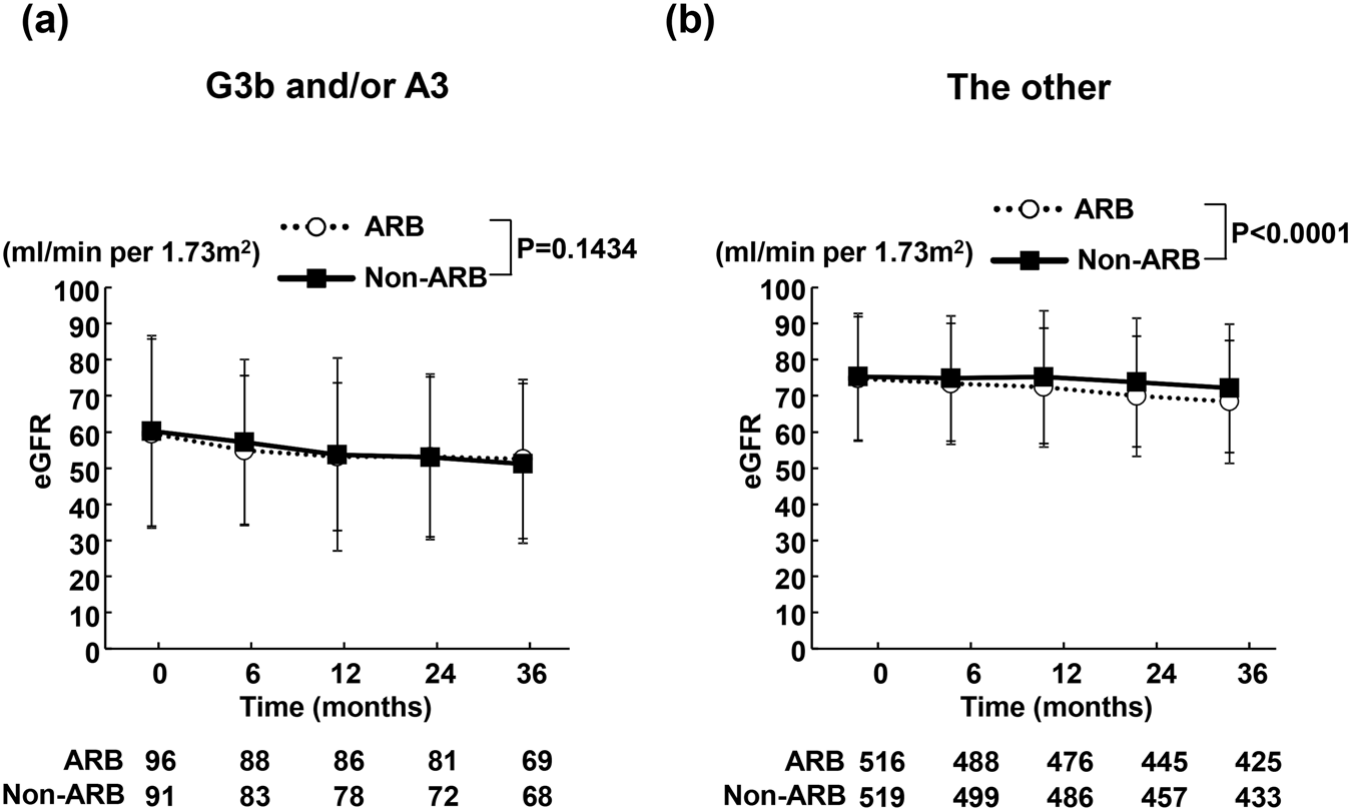
Time course of changes in estimated glomerular filtration rate (eGFR) in ARB and non-ARB groups in patients with eGFR of <45 ml/min per 1.73 m^2^ and/or UACR of ≥300 mg/g creatinine (**a**) and the other patients (**b**). Abbreviations used are the same as in Fig. 1. Values are mean ± SD.

As shown in Fig. 3(a), in patients with G3b and/or A3, no significant difference was noted regarding eGFR between ARB and non-ARB groups (p = 0.1434). On the contrary, eGFR in ARB group was significantly less than that in non-ARB group in the other patients (P < 0.0001) (Fig. 3(b)).

### Time course of change in plasma BNP during the follow-up period

**Figure 4:**
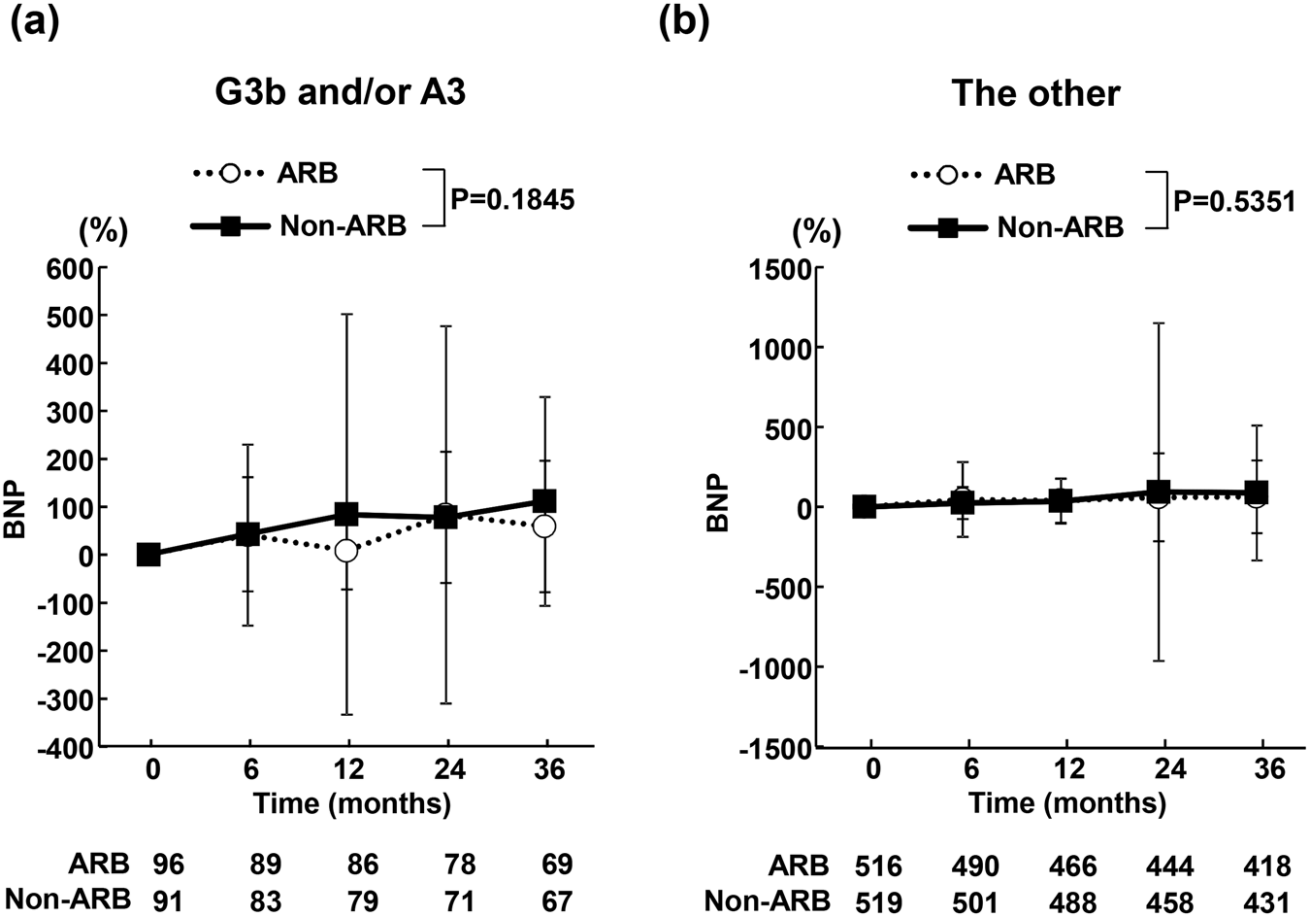
Time course of % changes in plasma brain natriuretic peptide (BNP) in ARB and non-ARB groups in patients with eGFR of <45 ml/min per 1.73 m^2^ and/or UACR of ≥300 mg/g creatinine (**a**) and the other patients (**b**). Abbreviations used are the same as in Fig. 1. Values are mean ± SD.

In both patients with G3b and/or A3 (Fig. 4(a)) and the other patients (Fig. 4(b)), plasma BNP levels were similar between ARB and non-ARB groups throughout the follow-up period.

### Time course of blood pressure during the follow-up period

**Figure 5:**
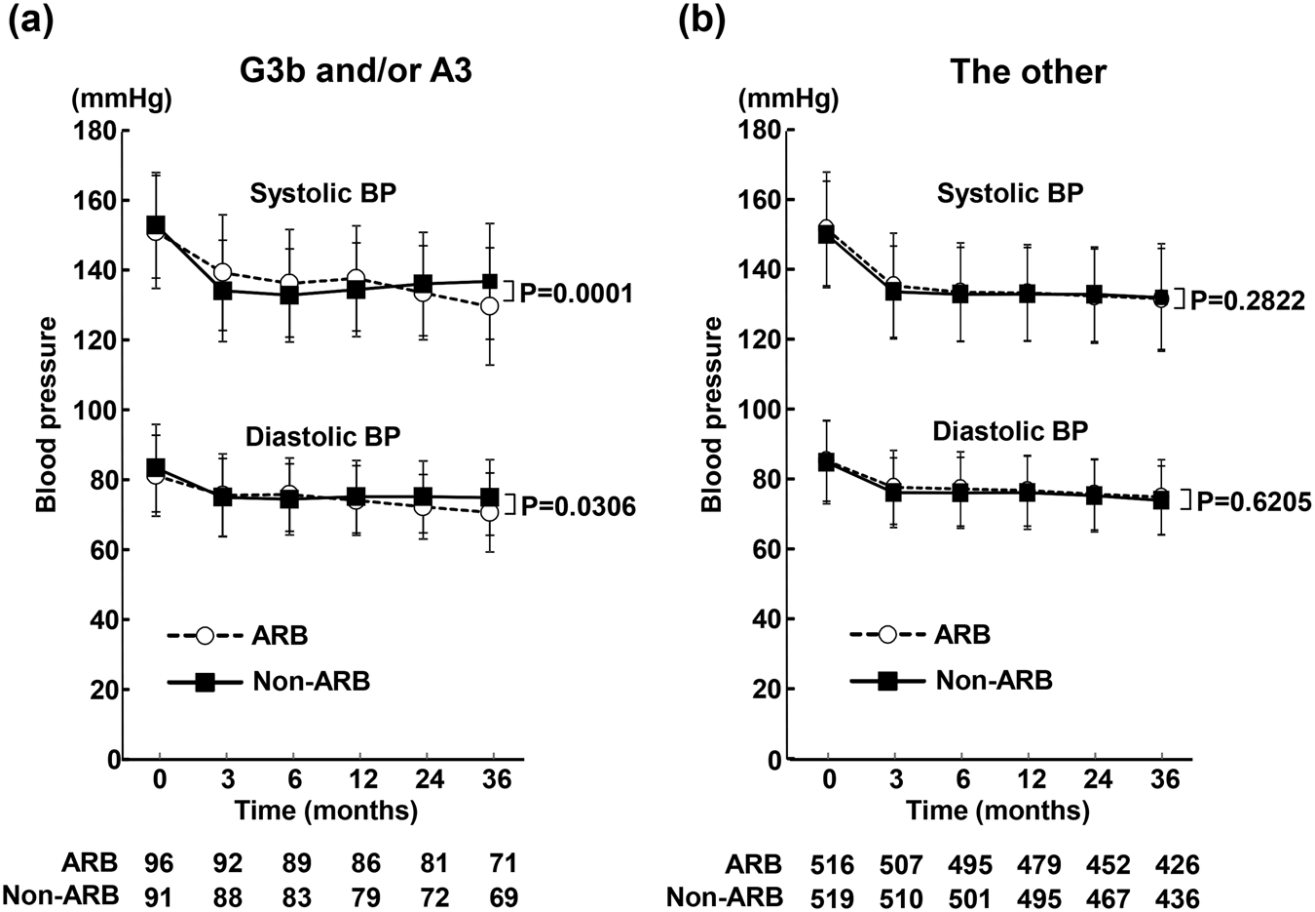
Time course of systolic and diastolic blood pressure (BP) in ARB and non-ARB groups in patients with eGFR of <45 ml/min per 1.73 m^2^ and/or UACR of ≥300 mg/g creatinine (**a**) and the other patients (**b**). Abbreviations used are the same as in Fig. 1. Values are mean ± SD.

Figure 5 and Table 5 indicate time course of blood pressure in each group of patients. In patients with G3b and/or A3, there was the significant difference between ARB and non-ARB groups regarding time course of systolic BP (P = 0.0001) and diastolic BP (P = 0.0306). At 3, 6, and 12 months, systolic BP in ARB group tended to be higher than that in non-ARB group. However, at all time points examined, the difference did not reach statistical significance regarding systolic or diastolic BP between ARB and non-ARB groups in patients with G3b and/or A3. Proportion of the patients achieved BP of <140/90 mmHg was 63.8% in ARB group and 69.3% in non-ARB group of patients with G3b and/or A3, and there was no significant difference between the groups (P = 0.4331).

**Table 5:** Time course of systolic and diastolic BP in patients with eGFR of <45 ml/min per 1.73 m^2^ and/or UACR of ≥300 mg/g creatinine or the other patients

	0 months	3 months	6 months	12 months	24 months	36 months
G3b and/or A3						
Systolic BP (mmHg)						
ARB group	151.0 ± 16.2	139.3 ± 16.6	136.2 ± 15.4	137.7 ± 15.1	133.5 ± 13.4	129.6 ± 16.8
Non-ARB group	152.9 ± 15.1	134.1 ± 14.5	132.8 ± 13.3	134.4 ± 13.4	136.1 ± 14.8	136.8 ± 16.6
Diastolic BP (mmHg)						
ARB group	81.2 ± 11.6	75.6 ± 11.8	75.8 ± 10.5	74.1 ± 9.9	72.3 ± 9.2	70.7 ± 11.3
Non-ARB group	83.4 ± 12.6	75.0 ± 11.1	74.5 ± 10.2	75.2 ± 10.4	75.2 ± 10.2	75.0 ± 10.8
The other						
Systolic BP (mmHg)						
ARB group	151.0 ± 16.3	134.7 ± 15.1	132.8 ± 14.1	132.7 ± 13.8	131.8 ± 13.5	131.0 ± 14.4
Non-ARB group	149.4 ± 15.3	133.0 ± 13.1	132.3 ± 13.5	132.4 ± 13.3	132.3 ± 13.5	131.4 ± 15.4
Diastolic BP (mmHg)						
ARB group	84.7 ± 11.6	77.1 ± 10.6	76.6 ± 10.6	76.1 ± 10.1	75.0 ± 10.1	74.3 ± 10.7
Non-ARB group	84.2 ± 11.9	75.5 ± 10.0	75.5 ± 10.2	75.6 ± 10.5	74.6 ± 10.3	73.3 ± 9.9

Abbreviations used are the same as in Table 2. Values are mean ± SD.

In the other patients, time course of systolic (P = 0.2822) and diastolic (P = 0.6205) BP was comparable between ARB and non-ARB groups during follow-up period. Proportion of the patients achieved BP of <140/90 mmHg was similar between ARB group and non-ARB group (76.5% vs 77.5%; P = 0.7061).

### Association of prognostic factors with cardiovascular and renal events

Table 6 shows the results of multivariable Cox regression analysis for overall patients, patients with G3b and/or A3, and the other patients. In overall patients, ARB allocation was not significantly associated with cardiovascular and renal events (P = 0.1520), while gender (P = 0.0353), previous CV disease (P < 0.0001), and previous diabetes (P < 0.0001) were significantly associated with cardiovascular and renal events. In patients with G3b and/or A3, ARB allocation (P = 0.0268) was significantly associated with cardiovascular and renal events, and previous diabetes (P = 0.0126) was also significantly associated with cardiovascular and renal events. In the other patients, ARB allocation (P = 08604) was not associated with cardiovascular and renal events, whereas age ≥ 65 years (P = 0.0165), previous CV disease (P = 0.0218), and previous diabetes (P = 0.0083) were significantly associated with cardiovascular and renal events.

**Table 6:** Adjusted hazard ratios of prognostic factor for cardiovascular and renal events.

	Overall patients		G3b and/or A3		The other	
	HR (95%CI)	P-value	HR (95%CI)	P-value	HR (95%CI)	P-value
ARB (+)	0.735 (0.483–1.120)	0.1520	0.437 (0.210–0.909)	0.0268	0.954 (0.565–1.612)	0.8604
Male gender	1.623 (1.034–2.548)	0.0353	2.097 (0.915–4.802)	0.0799	1.414 (0.817–2.449)	0.2159
Age ≧65 years	1.479 (0.929–2.355)	0.0990	0.847 (0.409–1.753)	0.6546	2.160 (1.151–4.055)	0.0165
Previous CV disease	3.235 (1.996–5.242)	<0.0001	3.221 (0.966–10.736)	0.0569	2.034 (1.109–3.730)	0.0218
Previous diabetes	3.678 (2.121–6.378)	<0.0001	3.433 (1.303–9.041)	0.0126	2.594 (1.279–5.263)	0.0083

Abbreviations: HR, hazard ratio; 95%CI, 95% confidence interval; CV, cardiovascular. Other abbreviations used are the same as in Table 2.

## Discussion

The present post-hoc analysis of the ATTEMP-CVD study was performed to examine the comparative effect of ARB (telmisartan)-based antihypertensive therapy and non-ARB antihypertensive therapy on the incidences of composite cardiovascular and renal events and biomarker changes in hypertensive patients with G3b and/or A3 (with at least one of eGFR of <45 ml/min per 1.73 m^2^ and UACR of ≥300 mg/g creatinine) or the other patients. The major findings of our subanalysis were as follows: (1)ARB-based antihypertensive therapy more reduced the incidence of composite cardiovascular and renal events than non-ARB therapy in patients with G3b and/or A3, while there was no significant difference in the incidence of cardiovascular and renal events between the two therapies in the other patients; (2) More benefit of ARB-based therapy over non-ARB therapy in prevention of cardiovascular and renal events in patients with G3b and/or A3 was associated with the significant decrease in UACR but not with blood pressure, eGFR or plasma BNP. The present subanalysis provided a new insight into antihypertensive therapeutic strategy in patients with G3b and/or A3 in terms of prevention of cardiovascular morbidity and mortality.

Solid evidence indicates that CKD is a risk factor for not only ESKD but also cardiovascular disease and death^3,4^. KDIGO guideline^4^ highly recommends that the definition and classification of CKD is determined by the level of both GFR and albuminuria, because decreased GFR and increased albuminuria levels both are significant risk factors for ESKD and cardiovascular disease and death independently of one another^6,8,9,11,12^. However, in most of previous clinical studies on CKD patients, the definition and classification of CKD patients have been determined based on only GFR category, and there is no sufficient evidence on randomized trials of CKD patients based on both GFR and albuminuria categories. The patients with eGFR of <45 ml/min per 1.73 m^2^ (G3b cut-off value) are at much higher risk for cardiovascular morbidity and mortality as well as for ESKD than the patients with eGFR of ≥45 ml/min per 1.73 m^2^ ^3–5^. Furthermore, regardless of GFR levels, patients with UACR of ≥300 mg/g creatinine (macroalbuminuria) have much higher risk for renal and cardiovascular diseases and death than patients without macroalbuminuria^3–5^. Therefore, CKD patients with at least one of eGFR of <45 ml/min per 1.73 m^2^ and macroalbuminuria (defined as G3b and/or A3, in the present study) are at higher risk regarding the prognosis than the other CKD patients, and randomized trial on such patients at higher risk should be more encouraged to develop more appropriate antihypertensive therapy for ESKD and cardiovascular outcomes. However, there is insufficient evidence for antihypertensive strategy in patients with G3b and/or A3. These findings encouraged us to examine the subgroup of the hypertensive patients enrolled in ATTEMPT-CVD study with at least one of eGFR of <45 ml/min per 1.73 m^2^ and UACR of ≥300 mg/g creatinine. In the present study, patients with G3b and/or A3 had much greater incidence of cardiovascular and renal events than the other subgroup, being in good agreement with the established evidence^3–5^.

The renoprotective effects beyond BP control of RAS blockers including ARBs and ACE inhibitors have been well established in CKD patients^14–21^ (particularly the patients with diabetic nephropathy). However, it remains to be elucidated whether RAS blockers are superior to other classes of antihypertensive drugs in CKD patients with G3b and/or A3 regarding prevention of cardiovascular events. The present analysis demonstrated that in hypertensive patients with G3b and/or A3, ARB therapy had less incidence of cardiovascular and renal events than non-ARB therapy, and allocation to ARB therapy was a significant independent prognostic factor of cardiovascular and renal events in such patients. Moreover, the incidence of doubling of serum creatinine was less in ARB group than in non-ARB group, further supporting the renoprotective effect of RAS blockade beyond BP control. Therefore, our study provided the evidence suggesting that ARB therapy may be more beneficial than non-ARB therapy in terms of prevention of cardiovascular and renal events in patients with G3b and/or A3. It is well established that BP reduction itself causes the prevention of CKD progression and cardiovascular disease and death^4,22–24^. However, in the present study, in patients with G3b and/or A3, BP during follow-up period tended to be higher in ARB group than in non-ARB group, although the difference did not reach statistical significance between ARB and non-ARB groups. Furthermore, there was no difference between ARB and non-ARB groups in the proportion of patients achieved BP of <140/90 mmHg. Therefore, it is unlikely that more benefit of ARB therapy over non-ARB therapy regarding prevention of cardiovascular and renal events in the patients with G3b and/or A3 might be attributed to blood pressure. Thus, BP-independent effects of ARB-based therapy seem to be responsible for less incidence of cardiovascular and renal events in ARB group than in non-ARB group of patients with G3b and/or A3.

We have previously reported the findings of the OSCAR study^25,26^, which is a prospective, randomized trial to investigate comparative effect of ARB (olmesartan 20 mg/day) plus CCB combination therapy versus high-dose ARB (olmesartan 40 mg/day) therapy in Japanese elderly hypertensive patients who had at least one of cardiovascular diseases or type 2 diabetes at baseline. In CKD subgroup analysis of the OSCAR study^25^, we defined CKD patients as those with eGFR of <60 ml/min per 1.73 m^2^ but did not measure UACR, and found that ARB plus CCB combination reduced the incidence of cardiovascular events more than high-dose ARB therapy in elderly hypertensive patients with CKD (with eGFR of <60 ml/min per 1.73 m^2^), and more benefit of ARB plus CCB combination in prevention of cardiovascular events was associated with more reduction of BP^25^. Therefore, our CKD subanalysis of the OSCAR study showed that the combination of ARB with CCB is superior to uptitration of ARB (high-dose ARB) in prevention of cardiovascular events in elderly hypertensive patients with eGFR of <60 ml/min per 1.73 m^2^, probably because of greater BP-lowering effect of ARB plus CCB combination. Of note, as previously described^1^, in the ARB group enrolled in the ATTEMPT-CVD, the percentage of patients prescribed CCB was only 28% at baseline and 37% at 36 months (the end of the study) and the percentage of patients prescribed β-blockers and diuretics was 13% and 14%, respectively, at 36 months. On the other hand, in non-ARB group of the ATTEMPT-CVD, the percentage of the patients prescribed CCB was 99% at baseline and 100% at 36 months, and other main prescribed antihypertensive drugs were β-blockers (25%), diuretics (20%),and ACE inhibitors (13%) at 36 months^1^. Therefore, differing from the findings of the CKD subanalysis of OSCAR study^25^, in the present study, ARB monotherapy itself seems to have more benefit in prevention of cardiovascular and renal events than non-RAS antihypertensive agents in hypertensive patients with G3b and/or A3. Future large-scale randomized trial on CKD patients with G3b and/or A3 addressing this issue is needed to elucidate our proposal.

It is suggested that UACR^16,27–30^ and eGFR changes are potentially useful for predicting future progression or prognosis of cardiovascular and renal diseases. In the present subanalysis, we also compared between ARB and non-ARB groups regarding the change of UACR and eGFR. Interestingly, in patients with G3b and/or A3, UACR during follow-up period was significantly less in ARB group than in non-ARB group, while ARB and non-ARB groups had similar eGFR changes. The improvement of UACR by ARB therapy, but not eGFR, was associated with less incidence of cardiovascular and renal events in patients with G3b and/or A3. On the other hand, in the other patients, no difference was noted between ARB and non-ARB groups regarding UACR, while ARB group has less eGFR than non-ARB group. Therefore, monitoring of albuminuria change may be more useful than that of GFR change in patients with G3b and/or A3.

Plasma BNP is also proposed as a potential biomarker predicting the prognosis of cardiovascular and renal diseases^31–33^. Importantly, in patients with G3b and/or A3, plasma BNP levels were significantly higher than in the other patients. However, the change of plasma BNP levels was comparable between ARB and non-ARB groups in patients with G3b and/or A3 or in the other patients. Therefore, the present study provided no evidence for the significance of plasma BNP as a marker predicting the prognosis of cardiovascular and renal events in CKD with G3b and/or A3.

ARB used in the present study was telmisartan. There are abundant experimental evidences indicating that differing from other ARBs, telmisartan is an ARB with partial PPAR-γ activity^34–37^. Therefore, it is an important question whether the benefit of telmisartan in prevention of cardiovascular events observed in this study was partially attributed to its PPAR-γ activity. However, in contrast to much experimental evidences, there is no clinical evidence indicating the significant role of PPAR-γ activity in telmisartan-induced organ protection. Furthermore, previous randomized double-blind placebo-controlled study^38^, which investigated the effect of telmisartan on PPAR-γ target genes CD36 and CD163 in patients with metabolic syndrome, showed that the activation of PPAR-γ target gene was not demonstrated by telmisartan at 80 mg (the highest dose used in our study) and partial activation of PPAR-γ target gene was observed only in 160 mg of telmisartan. Taken together, it is likely that cardiovascular and renal protective effect of telmisartan in this subanalysis was mediated by AT1 receptor blockade rather than PPAR-γ activity. Accumulating evidence^36,39^ supports the notion that AT1 receptor inhibition causes pleiotropic effects such as the amelioration of tissue oxidative stress and inflammation, glomerular hypertension, and cardiovascular remodelling. Therefore, the potential mechanisms underlying the benefit of telmisartan demonstrated in this subanalysis seem to be derived from the above mentioned pleiotropic effects^36,39^ induced by AT1 receptor inhibition. Future randomized study is required to elucidate the precise mechanism of cardiovascular protection by ARBs.

### Study limitation

There are several study limitations in our subanalysis. First, the number of patients with G3b and/or A3 was small and our present findings are hypothesis generating. Such insufficient sample size seems to account for the finding that the treatment-subgroup interaction did not reach statistical significance. Large-scale prospective randomized trial on patients with G3b and/or A3 is required to define our present findings. Second, our present subanalysis is post-hoc analysis. However, we believe that our present proposal of categorization of the patients based on G3b cut-off value (eGFR of 45 ml/min per 1.73 m^2^) and A3 cut-off value (UACR of 300 mg/g) has the significant clinical implication, because the patients with G3b and/or A3 are at higher risk regarding prognosis. Furthermore, it provides important information for clinical decision making in terms of the therapeutic strategy for renal and cardiovascular outcomes. Finally, the present subanalysis did not allow us to determine whether more reduction of UACR by ARB therapy in patients with G3b and/or A3 was directly linked to fewer incidences of cardiovascular and renal events by ARB therapy. Future study demonstrating the direct association of UACR change with incidence of cardiovascular and renal events should be designed.

In conclusion, our present subanalysis provided the evidence suggesting that ARB-based antihypertensive therapy may have the benefit in prevention of cardiovascular and renal events in hypertensive patients with G3b and/or A3 (with either eGFR of <45 ml/min per 1.73 m^2^ or UACR of ≥300 mg/g creatinine, or both). Our subanalysis provides a new insight into antihypertensive strategies for CKD. However, large-scale randomized trial is required to define our hypothesis generating findings.

## Methods

### Study design and treatment protocol

The detail of study design and treatment protocol of the ATTMEPT-CVD study has been previously reported^2^. This trial was registered with ClinicalTrials.gov number NCT01075698. In brief, ATTEMPT-CVD study is a multicentre, prospective, randomised, open-label, active-controlled trial with blinded end-point assessment of 1,228 hypertensive patients aged 40 to 80 years who had at least one of cardiovascular risk factors (type 2 diabetes, renal dysfunction, cerebrovascular disease, cardiac disease or peripheral artery disease). Full inclusion and exclusion criteria are described in our previous papers^1,2^. The study protocol was in agreement with the ethics committee guidelines of our institution and complied with the Declaration of Helsinki. The institutional review board of each participating hospital approved this trial, and written informed consent was obtained from each patient. The study protocol was approved by the ethics committee of Kumamoto University.

The eligible patients were randomly assigned in a 1:1 ratio by computer-generated stratified randomization sequence were stratified for age, sex, history of cardiovascular events, history of diabetes mellitus, and usage of an ACE inhibitor and was allocated (1) treatment with telmisartan, an ARB (ARB group) or (2) treatment with antihypertensive drugs except for ARB (non-ARB group). Patients and study investigators were not masked for treatment allocation. After completion of registration and allocation, administration of telmisartan at the indicated low (20 mg/day) or middle (40 mg/day) dose was started in the ARB group. The dose level of telmisartan could be increased to the middle or highest dose (80 mg/day) by the physician’s decision in patients who did not achieve the target blood pressure. In the non-ARB group, an antihypertensive drug except for ARB was started.

At study registration and after 3, 6, 12, 24 and 36 months, a physician examined the survey items including discontinuation/dropout, occurrence of any cardiovascular events, and occurrence of any adverse events. At 6, 12, 24 and 36 months, drug compliance, concomitant drugs, concurrent therapies, physical findings, and laboratory tests and biomarkers including urinary albumin/creatinine ratio (UACR), plasma BNP levels, serum high-sensitivity C-reactive protein (hsCRP) levels, urinary 8-hydroxy-deoxy-guanosine (8-OHdG), serum adiponectin and high-molecular weight adiponectin levels and estimated glomerular filtration rate (eGFR) were examined.

### Endpoints

The primary endpoints of ATTMEPT-CVD study were changes in UACR and in plasma BNP levels from baseline^2^. The secondary endpoint was the time to the first occurrence of composite cardiovascular and renal events consisting of cerebrovascular events, cardiac events, peripheral arterial events, complication of diabetes, and aggravation of renal function^2^. In addition, changes in eGFR, hsCRP, urinary 8-OHdG, serum adiponectin, and serum high-molecular weight adiponectin were also the secondary endpoints.

### Subgroup analysis according to eGFR and UACR categories at baseline

In the present analysis, the patients enrolled in the ATTEMPT-CVD study were divided into two subgroups by applying G3b cut-off value (GFR of 45 ml/min per 1.73 m^2^) and albuminuria A3 cut-off value (UACR of 300 mg/g creatinine). One subgroup was the patients with at least one of eGFR of <45 ml/min per 1.73 m^2^ and UACR of ≥300 mg/g creatinine (macroalbuminuria) at baseline, which were defined as patients with G3b and/or A3 (see the category enclosed by a thick line in Table 1). The other subgroup was patients with both eGFR of ≥45 ml/min per 1.73 m^2^ and UACR of <300 mg/g creatinine at baseline, which were defined as the other patients.

### Statistical analysis

Sample size and power of the study were estimated as previously described^1,2^. All analyses were performed on the intention-to-treat population. All randomized patients with at least one on-treatment observation of laboratory data and safety information at that point were included in efficacy and safety analyses. Subjects who withdrew consent were excluded.

As for cardiovascular and renal events, time to first event curves were estimated by the Kaplan-Meier method and the log-rank test was used to show the differences between ARB and non-ARB groups. Using Cox proportional hazard model, the hazard ratio (HR) of the ARB group to the non-ARB group and its 95% confidence interval (CI) were calculated. To estimate the heterogeneity of the HR for the subgroup according to baseline eGFR and UACR, the interaction between treatment groups and the subgroup according to eGFR and UACR was assessed using the interaction terms in a stratified Cox proportional-hazards model. Repeated measures analysis of variance was used to compare between ARB and non-ARB groups for time course of blood pressure during the follow-up period, and compared using the unpaired t-test adjusted by Holm’s method to avoid multiplicity at multiple time points. Multivariable Cox proportional hazards analysis was performed to determine the association of each prognostic factor with the incidence of cardiovascular and renal events adjusted for the following covariate: sex, age, treatment group, baseline cardiovascular diseases, baseline diabetes.

The changes in UACR, plasma BNP, or eGFR were compared between ARB and non-ARB groups and analysed by repeated-measure analysis of variance (ANOVA) with measurement time points as repetition.

Windows SAS Version 9.2 and subsequent versions were used as the statistical analysis software. P-values of less than 0.05 were considered statistically significant.

### Data availability

The datasets generated during and/or analysed during the current study are available from the corresponding author on reasonable request.

## Electronic supplementary material

Supplementary Information
