# Supplementary material for: Cardiovascular and renal protective role of angiotensin blockade in hypertension with advanced CKD: a subgroup analysis of ATTEMPT-CVD randomized trial
Source: Sci Rep. 2018 Feb 16;8:3150. doi: 10.1038/s41598-018-20874-4 (PMC5816600; doi:10.1038/s41598-018-20874-4)
Supplement: Supplementary file 1 — Supplementary Information [file 41598_2018_20874_MOESM1_ESM.docx]

**Appendices**

A Trial of Telmisartan Prevention of Cardiovascular Diseases (ATTEMPT-CVD) Investigators

Steering Committee: Hisao Ogawa, Shokei Kim-Mitsuyama, Koichi Node, Hideaki Jinnouchi, and Taiji Sekigami

Committee on Validation of Data and Events: Sohichi Uekihara, Yoichiro Hashimoto, and Shuichi Oshima

Safety Monitoring Board: Kazuteru Fujimoto, Toshiro Yonehara, and, Teruyuki Hirano

Study Statistician: Kunihiko Matsui

Independent Data Management Center: Kaori Harada, Nobue Ueno, Yoshiko Terasaki, Mami Okamoto, Kanae Kanamori, Naoko Oka, Nami Matsuo, and, Kaori Sakaguchi

Investigators: Hirofumi Soejima, Osamu Yasuda, Megumi Yamamuro, Eiichiro Yamamoto, Yoichi Hanaoka, Hirofumi Kan, Tomio Jinnouchi, Syuichi Matsuo, Yutaka Wakasa, Naoto Yokota, Kazuo Matsunaga, Takatoshi Otonari, Toshihiko Sakanashi, Masanori Nishiyama, Hiroo Miyagi, Satoru Suzuki, Shukuko Ebihara, Masayasu Yamamoto, Ryo Fukami, Yoshimi Ohshima, Masahisa Ori, Junji Shibata, Daisuke Fujimatsu, Kouichi Mizobe, Michio Mizobe, Masato Nishimura, Shigeki Gondo, Juniti Miyata, Natsuki Nakamura, Koichi Kikuta, Yasuhiro Sakamoto, Shirou Mimori, Takashi Ono, Kunihiro Ohmori, Hirofumi Naitoh, Yasushi Suzuki, Shinichi Hirota, Kohji Honjo, Yoji Yamasaki, Kazushi Serikawa, Hideo Ikeda, Kazuo Ibaraki, Shigemasa Hashimoto, Sueo Momosaki, Satoshi Abe, Motoki Yoshinari, Kouji Sasaki, Masamitsu Toihata, Eiko Kobayashi, Takuo Ogawa, Shu Konno, Yuji Tanaka, Naoki Yamamoto, Kazuya Shigenobu, Hiromi Fujii, Kazunori Murakami, Osamu Doi, Mari Kashiwabara, Kiyoharu Itoh, Fumihiro Oishi, Azusa Ikegami, Takao Mishima, Jouji Yamauchi, Shoichi Kitano, Hiroshi Haruguchi, Tomohiro Katsuya, Shunsuke Take, Sadao Nakajima, Haruaki Uchiyama, Yoshihiro Yamada, Munetaka Yamaguchi, Eiichiro Honda, Shinichi Uemura, Yasuhiro Morikami, Teruhiko Ito, Yoko Oe, Takashi Fukunaga, Yoshikuni Haraguchi, Masahiko Tsuji, Shigeru Kiyama, Kazuo Kuroki, Shigeya Tanaka, Kenichi Ashihara, Kenzo Motoki, Kenya Kusunose, Junko Hotchi, Hisashi Shimono, Kenichiro Iimori, Hisashi Watanabe, Fujio Yoshida, Kazuo Machii, Hiroshi Yamamoto, Kazuhisa Kodama, Atsushi Hiwatashi, Sumio Komatsu, Nakayasu Wake, Takashi Ise, Senshu Hifumi, Jun Ohbayashi, Shojiro Naomi, Yutaka Horio, Ken Iwai, Toshiro Matsunaga, Hiroshi Sakamoto, Yoshinobu Morikawa, Eisaku Harada, Yoshinobu Murasato, Masataka Horiuchi, Naomi Sugahara, Soichi Honda, Shingo Shoji, Masanori Shida, Toshinori Utsunomiya, Takao Baba, Moronari Matsunaga, Kazuyoshi Noda, Yoichi Setoguchi, Kohei Yamaguchi, Masayoshi Koga, Masashi Sakai, Yoshihisa Tamazaki, Genjiro Sato, Takanobu Nakajima, Masayuki Otani, Atsuyuki Wada, Jun Yamagami, Shinji Tamaki, Mitsuo Morita, Masako Waki, Tsukasa Katsuki, Kenji Sadamatsu, Yasuaki Koga, Shinsuke Tsuji, Yoshito Inobe, Kenji Misumi, Hiroto Okubo, Hirofumi Matsuda, Koichiro Kataoka, Kyoji Takaoka, Osamu Hashiguchi, Tomohiro Sawada, Tomonori Kanazawa, Kazuhiko Matsuo, Kunio Idegami, Eiichiro Tanaka, Yoshio Horita, Satoru Horita, Akira Maki, Koji Sasaki, Tomoki Nakamura, Masaru Doi, Katsunori Kawamitsu, Hirofumi Maeda, Takaoki Otsuka, Hiroyuki Tanaka, Takamasa Iwasawa, Kensei Hayashida, Hiroyuki Tanaka, Katuya Oosima, Yasuji Doi, Yasuhiro Nishiyama, Masashi Ikushima, Toru Kinugawa, Kaname Akioka, Yoko Anbe, Hiroaki Kono, Yasuhiro Sasaki, Mario Yamaki, Takashi Honda, Atsushi Sato, Takahiro Hayashi, Yoshihiro Kimura, Aya Shiraki, Shunichi Sugimoto, Yasuharu Kodama, Hideki Wakamatsu, Shiho Enomoto, Yoshiyasu Yamatsu, Sadaaki Okamoto, Hiroaki Hosokawa, Kouji Maeno, Yohsuke Hanaoka, Hisanori Kanazawa, Tadasuke Chitose, Taishi Nakamura, Junichi Matsubara, Shinsuke Hanatani, Kenichi Tsujita, Satoshi Araki, Hiroaki Kusaka, Kenshi Yamanaga, Kensuke Toyama
